# 128 Use of Sublingual Dexmedetomidine for Burn Wound Care

**DOI:** 10.1093/jbcr/irae036.127

**Published:** 2024-04-17

**Authors:** Laura K Madsen, Scott W Mueller, Jeffrey Glasheen, Cameron Gibson, Arek J Wiktor

**Affiliations:** University of Colorado Hospital, Denver, Colorado; UCHealth and University of Colorado School of Pharmacy, Aurora, CO; Institute for Healthcare Quality, Safety and Efficiency, Aurora, Colorado; University of Colorado, Aurora, CO; University of Colorado Hospital, Aurora, CO; University of Colorado Hospital, Denver, Colorado; UCHealth and University of Colorado School of Pharmacy, Aurora, CO; Institute for Healthcare Quality, Safety and Efficiency, Aurora, Colorado; University of Colorado, Aurora, CO; University of Colorado Hospital, Aurora, CO; University of Colorado Hospital, Denver, Colorado; UCHealth and University of Colorado School of Pharmacy, Aurora, CO; Institute for Healthcare Quality, Safety and Efficiency, Aurora, Colorado; University of Colorado, Aurora, CO; University of Colorado Hospital, Aurora, CO; University of Colorado Hospital, Denver, Colorado; UCHealth and University of Colorado School of Pharmacy, Aurora, CO; Institute for Healthcare Quality, Safety and Efficiency, Aurora, Colorado; University of Colorado, Aurora, CO; University of Colorado Hospital, Aurora, CO; University of Colorado Hospital, Denver, Colorado; UCHealth and University of Colorado School of Pharmacy, Aurora, CO; Institute for Healthcare Quality, Safety and Efficiency, Aurora, Colorado; University of Colorado, Aurora, CO; University of Colorado Hospital, Aurora, CO

## Abstract

**Introduction:**

Dexmedetomidine is a sedative with advantageous analgesic properties. Sublingual administration (SL DEX) has been used successfully in various populations. Burn patients experience high levels of pain and anxiety, often difficult to treat with conventional agents. The aim of this quality improvement project was to evaluate the safety and efficacy of SL DEX when given prior to burn wound care.

**Methods:**

This was a pre-post paired analysis at an ABA verified burn center. Patients who had inadequate pain control during burn wound care utilizing our standard opiate/benzodiazepine/ketamine methods were eligible for SL DEX (2 mcg/kg) 30 minutes prior to wound care provided a baseline heartrate (HR) and mean arterial pressure (MAP) were 65 or greater. Rescue analgesia/anxiolysis, pain/sedation scores (RASS), and hemodynamic variables were the primary outcome measures. Each SL DEX session was matched to an equal amount of previous wound care sessions and averaged overall for efficacy and safety analysis.

**Results:**

A total of 20 patients (79% male) received SL DEX. A total of 86 wound care sessions were analyzed, 43 with SL DEX administrations and 43 without. The median age and TBSA burned were 40 years and 10% (IQR 4.5-22.5), Prior to SL DEX intervention, all patients had already received an oral opiate + lorazepam, 80% oral ketamine, 30% IV DEX, and 30% IV ketamine. Pre-wound care medications and clinical variables were similar between the groups. Non-exclusive indications for SL DEX included: escalation of wound care analgesia 65%, step-down therapy from IV DEX in 35%, pre-emptive for donor site dressing take down in 25%. SL DEX was associated with a significant reduction of average fentanyl administered (166mcg vs 134mcg; P=0.039), average pain and maximum pain scores (7.4 vs 5.9, p=0.042 and 8.6 vs 6.7, p=0.002; respectively), and a numerically less midazolam and IV ketamine (1.37mg vs 1.08mg, p=0.076 and 65.6mg vs 29.7mg, p=0.123). Intra-wound care RASS were not significantly different with SL DEX (matched mean difference -0.06, p>0.8). MAP and HR variables (maximum and minimum) were not different prior to, during, or following wound care.

**Conclusions:**

SL DEX was associated with decreased rescue analgesia requirements and lower patient reported pain scores without causing adverse hemodynamic effects in the difficult to treat burn population. A larger controlled trial is required to elucidate the efficacy and safety of SL DEX for wound care tolerance in the burn population.

**Applicability of Research to Practice:**

SL DEX provides a unique route of an adjunctive agent to improve wound care tolerability, pain, and sedation in difficult to treat patients. It may be a viable alternative to IV DEX for wound care de-escalation.

